# LAT1 expression in head and neck cancer: a prognostic biomarker with potential relevance for BNCT

**DOI:** 10.3389/fonc.2025.1678011

**Published:** 2025-12-19

**Authors:** Stefano Cavalieri, Imperia Nuzzolese, Deborah Lenoci, Marta Lucchetta, Marica Ficorilli, Ester Orlandi, Loris De Cecco, Lisa Licitra

**Affiliations:** 1 Head and Neck Medical Oncology Department, Fondazione IRCCS Istituto Nazionale dei Tumori, Milan, Italy; 2 Department of Oncology and Hemato-Oncology, University of Milan, Milan, Italy; 3 Integrated Biology of Rare Tumors, Department of Experimental Oncology, Fondazione IRCCS Istituto Nazionale dei Tumori, Milan, Italy; 4 Department of Clinical, Surgical, Diagnostic, and Pediatric Sciences, University of Pavia, Pavia, Italy; 5 Radiation Oncology Clinical Department, National Center for Oncological Hadron Therapy (CNAO), Pavia, Italy

**Keywords:** LAT1, HNSCC, gene expression profiling, BNCT, prognosis

## Abstract

**Purpose:**

The increased expression of LAT1, an amino acid transporter, in cancer cells makes boronophenylalanine (BPA) uptake higher in cancer *vs*. healthy tissues: a high LAT1 expression on cancer cells implies a higher sensitivity to boron neutron capture therapy (BNCT). We explored the LAT1 expression in a cohort of head and neck cancer (HNSCC) patients, stratifying them according to a previously published transcriptomic 6-cluster model.

**Methods:**

We analyzed 100 HNSCC patients treated with multimodal treatments including radiotherapy. Transcriptomics of primary tumor specimens was obtained by Affymetrix ClariomD chips and processed using the Transcriptome Analysis Console Software (ThermoFisher). We retrieved normalized and log2 LAT1 from the data matrix. Data were used to analyze: i) the distribution by anatomical subsites and transcriptomic subtypes (assessed by Kruskal-Wallis test); ii) overall survival (OS).

**Results:**

LAT1 expression was high (>2.83) in 13% of cases. At median follow-up of 64.44 months (95% CI: 54.24-66.91), overall median OS was 94.24 months, 22.99 months (95% CI 14.31-NR) in patients with high LAT1 *vs*. 94.24 months (95% CI 65.1-NR) in those with low LAT1. LAT1 expression did not differ significantly among HNSCC primary sites. Among GE clusters, the highest LAT1 expression was observed in those with the worst prognosis, the lowest in the immune-reactive one (p=.000028).

**Conclusion:**

High LAT1 expression has a negative prognostic role and is associated with transcriptomic clusters with unfavorable and radioresistant biologic features. These results justify the use of BNCT in radioresistant HNSCCs and may guide patient selection for future clinical studies with BNCT.

## 1 Introduction

The L-type amino acid transporter 1 (LAT1), encoded by the SLC7A5 gene, is a sodium-independent transporter for large neutral amino acids playing a central role in tumor metabolism. Its overexpression has been extensively documented in solid tumors, where it supports anabolic growth and correlates with poor outcomes. A meta-analysis of 35 studies involving over 4,500 patients demonstrated that high LAT1 expression predicts inferior overall survival (HR = 1.848), disease-free survival (HR = 1.923), and progression-free survival (HR = 1.345) (1). Similar associations have been observed in non-small cell lung cancer, pancreatic cancer, and biliary tract cancers (1), and more recently in high-grade subtypes of lung adenocarcinoma (2). This extensive evidence supports LAT1 as a broadly relevant prognostic marker in oncology, independent of tumor origin.

**Figure 1:**
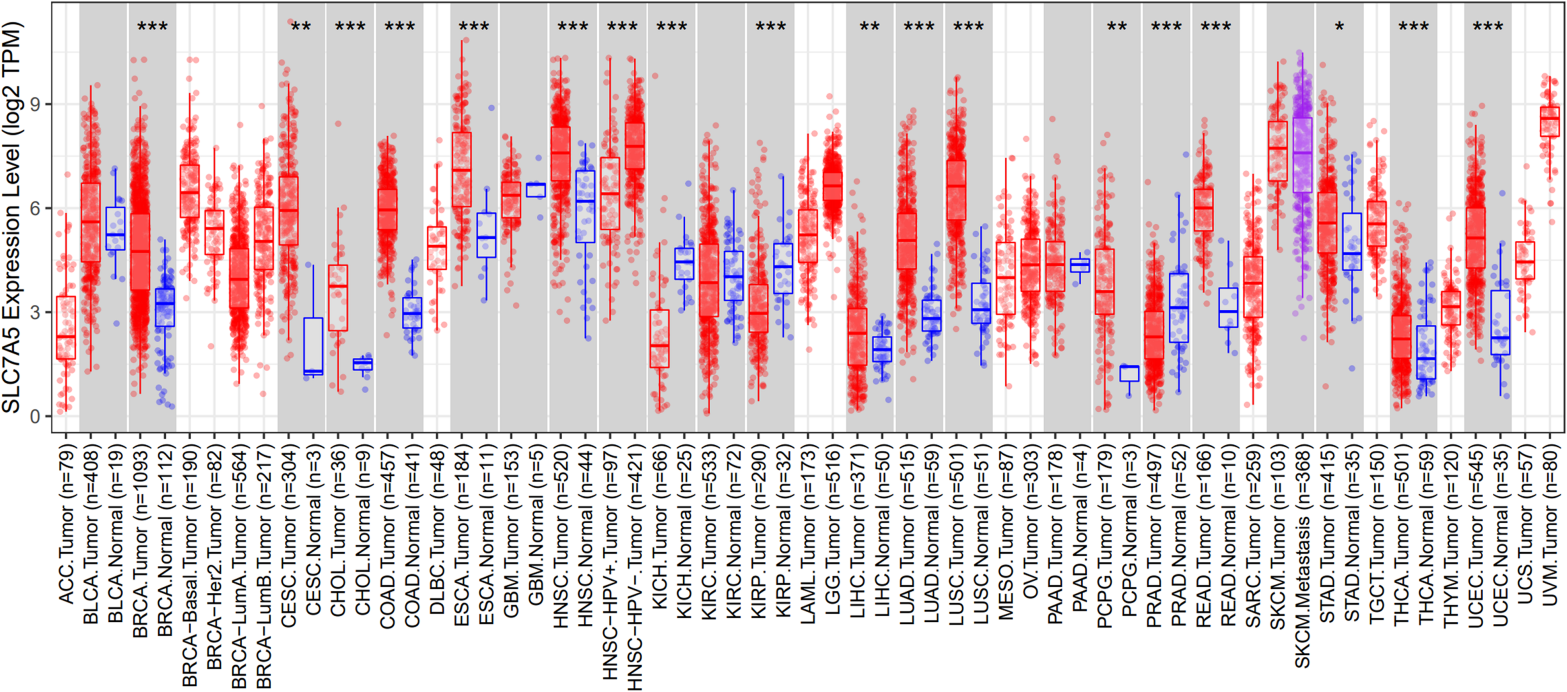
Pan-cancer LAT1 expression in TCGA

Among the various cancer types analyzed in the TCGA, head and neck squamous cell carcinoma (HNSCC) exhibited particularly elevated patterns of LAT1 gene expression compared to the other tumor types (3, 4). In addition, in TCGA, HNSCC tumors demonstrated significantly increased expression levels of LAT1 compared to normal tissue counterparts (**Figure 1**).

HNSCC is a biologically and clinically heterogeneous group of diseases often treated with radiotherapy as part of a curative multimodal strategy (5, 6). In recent years, innovations in radiation delivery have included advanced techniques such as boron neutron capture therapy (BNCT), which relies on high linear energy transfer (LET) particles produced by the nuclear reaction between thermal neutrons and boron-10, selectively transported into tumor cells by boron-containing agents such as boronophenylalanine (BPA). BNCT is entering the therapeutical arsenal for aggressive cancers that are not responsive to conventional therapies as a promising highly selective particle radiotherapy, including locally recurrent HN cancer (7).

In this context, LAT1 facilitates BPA uptake, so it has gained attention as a prognostic biomarker and a potential target for both imaging and therapy (8). However, its role in HNSCC and its potential for patient stratification in BNCT remain underexplored. Moreover, the biological context of LAT1 expression in HNSCC, and its association with known radioresistant tumor phenotypes, is not fully characterized. LAT1 expression in a well-annotated cohort of HNSCC patients and assesses its prognostic significance and relevance for BNCT candidate selection.

## 2 Methods

We retrospectively analyzed gene expression data from 100 patients with non-metastatic, loco-regionally advanced HNSCC treated with curative intent at the Fondazione IRCCS Istituto Nazionale dei Tumori, Milan, Italy, and included in the BD2Decide project (9). All patients had available primary tumor samples and full clinical data, as previously reported (10).

RNA was extracted from formalin-fixed, paraffin-embedded (FFPE) tumor tissue and analyzed using Clariom D arrays (Thermo Fisher Scientific, Waltham, MA, USA). Data were processed with Transcriptome Analysis Console software (Thermo Fisher Scientific). Log2-normalized expression values for LAT1 (SLC7A5) were extracted from the expression matrix.

LAT1 expression was analyzed across anatomical subsites and transcriptomic clusters previously defined (5). The Kruskal-Wallis test was used to evaluate expression differences. Patients were stratified as LAT1-high versus LAT1-low using the 85th percentile (cut-off = 2.83). Overall survival (OS) was estimated using the Kaplan-Meier method, and survival differences were tested with the log-rank test. Hazard ratios (HRs) were calculated using Cox proportional hazards modeling. Gene Set Enrichment Analysis (GSEA) (11) was performed to identify the deregulated biological pathways in the LAT1-high versus LAT1-low patients. Statistical analyses were performed using R (v4.2.0). Additional validation analyses in independent cohorts and considerations about potential functional *in vitro* explorations are reported in the **Supplementary Material**.

## 3 Results

**Figure 2:**
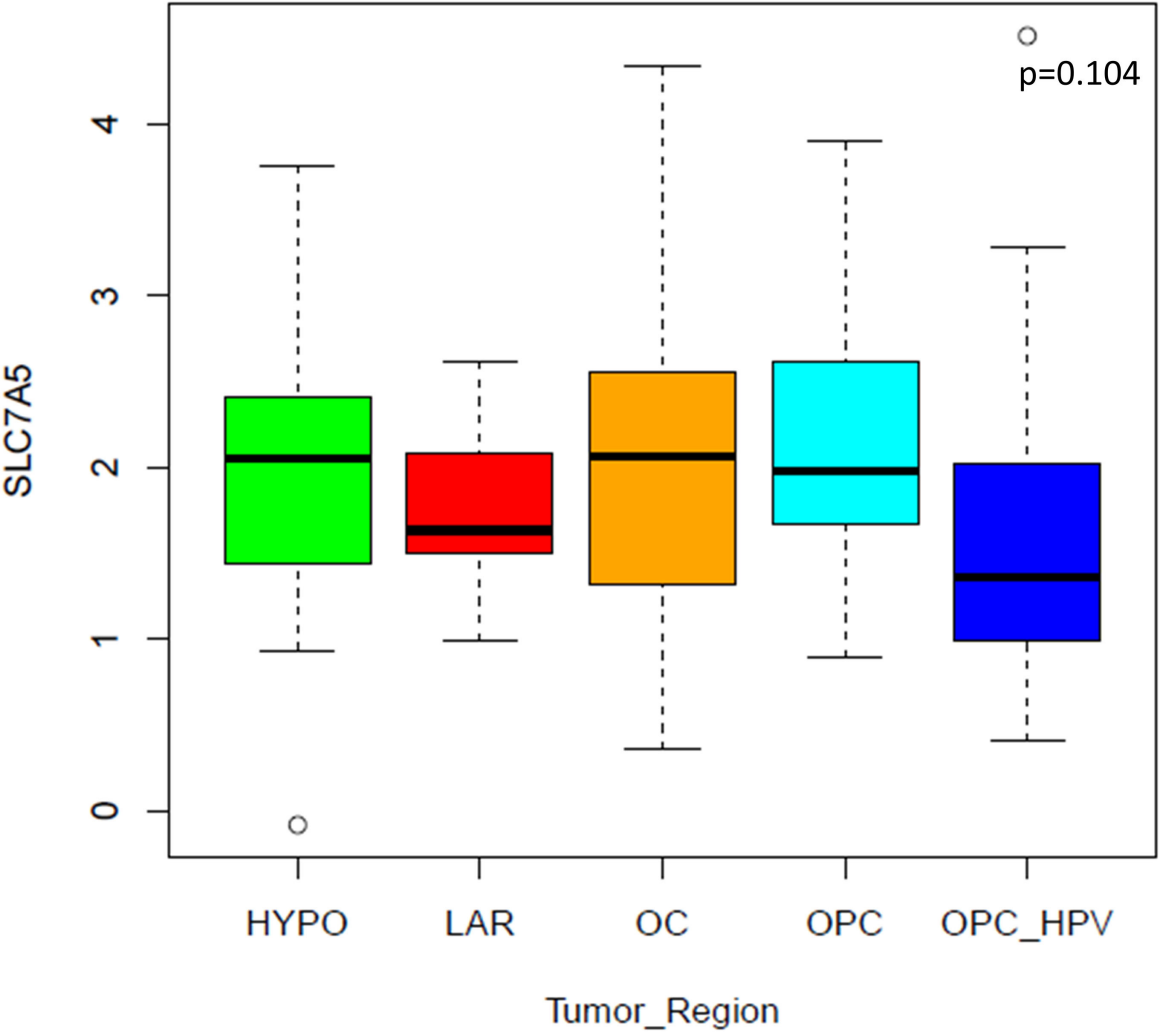
LAT1 expression according to HNSCC primary site. Legend: HYPO (hypopharynx), LAR (larynx), OC (oral cavity), OPC (HPV-negative oropharynx), OPC_HPV (HPV-related oropharynx)

We examined LAT1 expression in our cohort including 100 patients, evenly distributed by primary site (n = 20 each: oral cavity, HPV-positive oropharynx, HPV-negative oropharynx, hypopharynx, larynx). In our cohort, 24 were women, median age was 60 years (range 21-80) (10). LAT1 expression did not significantly vary across anatomical subsites (p = 0.104). Although no statistically significant difference was found, a numerically higher value of median LAT1 was found in hypopharynx, HPV-negative oropharynx, and oral cavity cancer, compared to laryngeal and HPV-positive oropharyngeal carcinomas (**Figure 2**).

**Figure 3:**
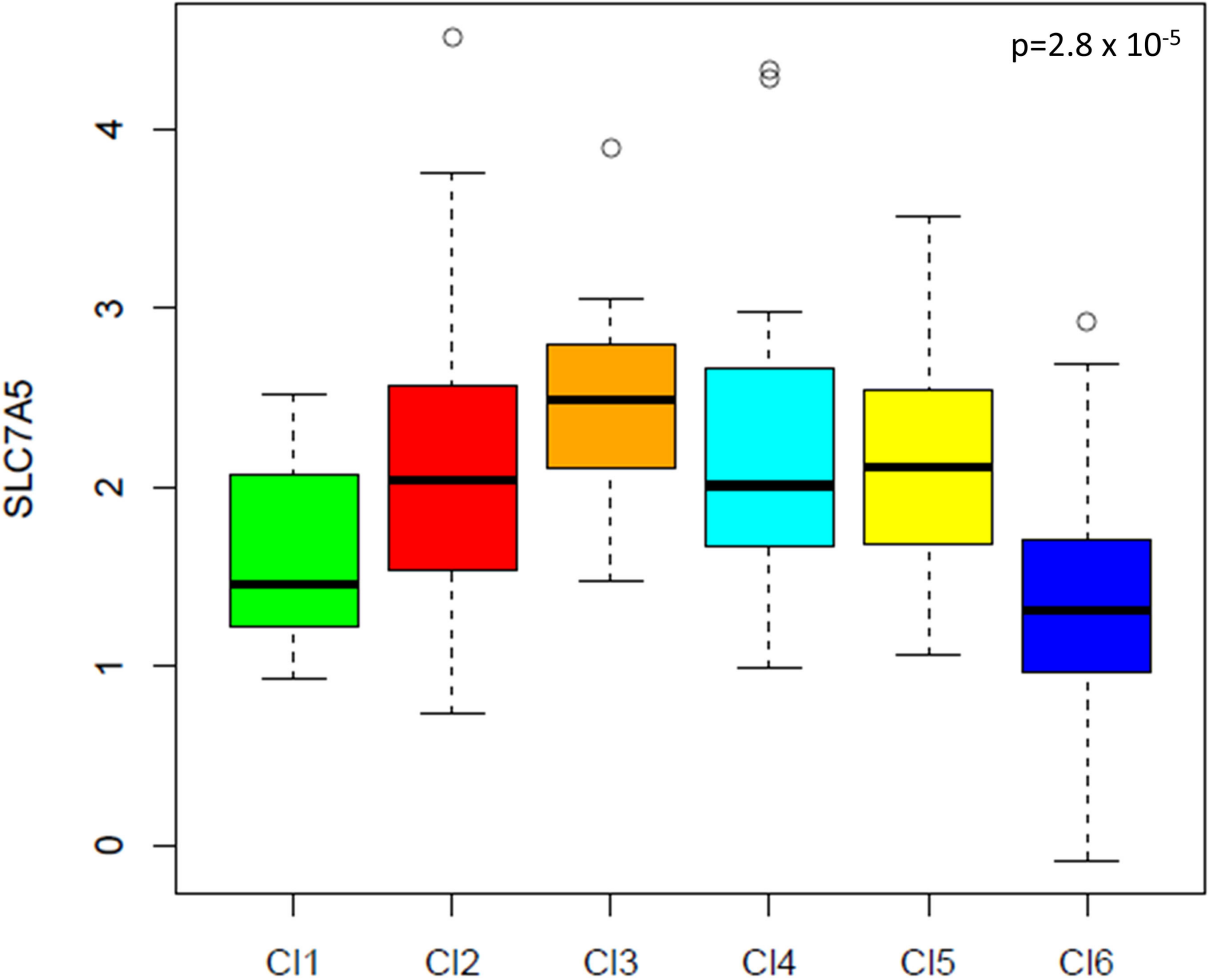
LAT1 expression based on GE profiling. Legend: Cl1, HPV-like; Cl2, mesenchymal; Cl3, hypoxia; Cl4, defense response; Cl5, classical; Cl6, immune reactive

Significant differences in LAT1 expression were observed across gene expression clusters (p = 2.8 x 10^-5, **Figure 3**). The highest expression was found in mesenchymal (Cluster 2) and hypoxic (Cluster 3) subtypes, associated with poor prognosis and radioresistance; the lowest expression was in the immune-reactive subtype (Cluster 6).

**Figure 4:**
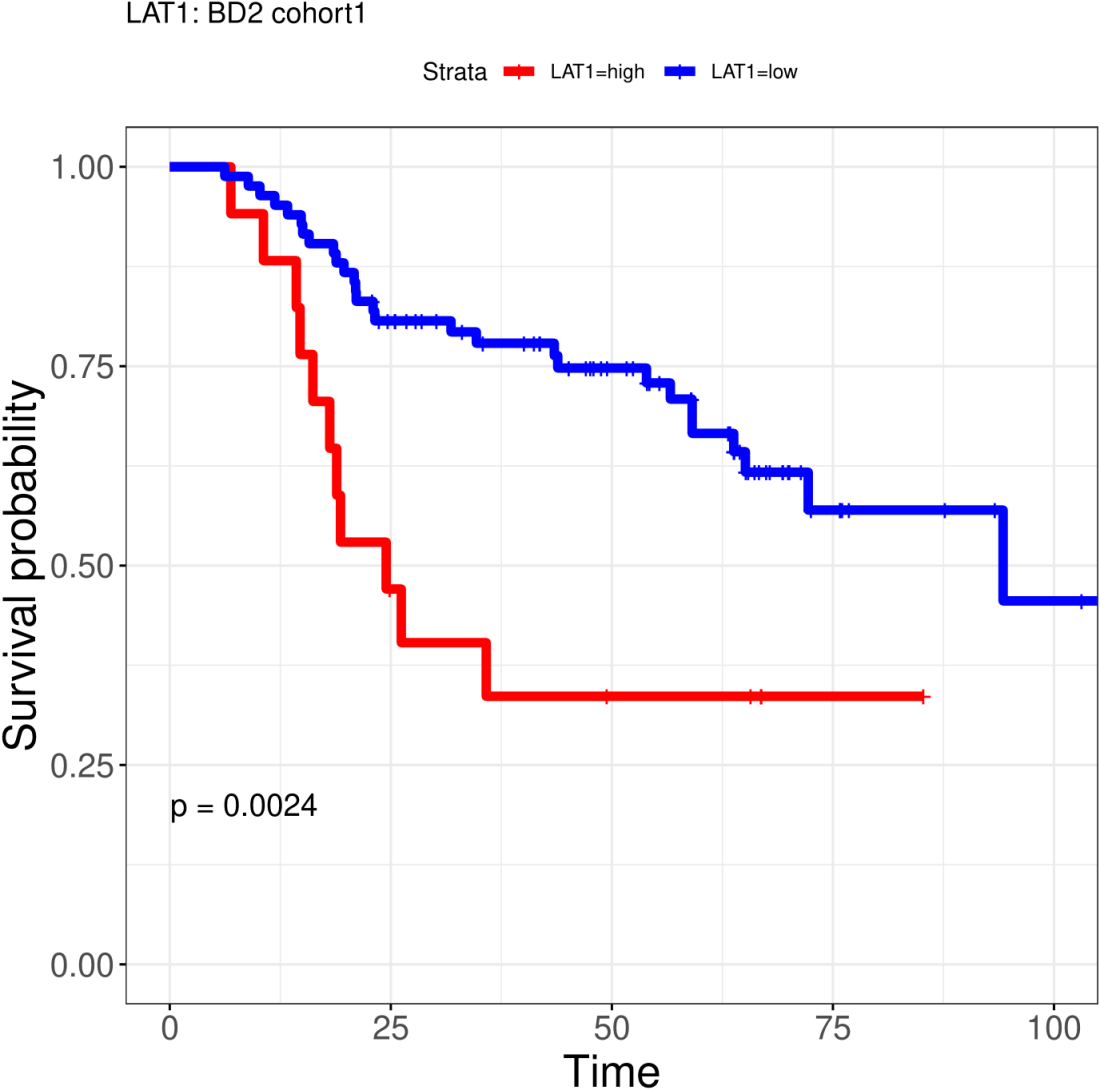
Prognostic value of LAT1 expression. Expression of LAT1 was associated to overall survival

LAT1-high patients (n = 13) had significantly shorter OS compared to LAT1-low patients (n = 87). Median OS was 22.99 months (95% CI: 14.31-NR) versus 94.24 months (95% CI: 65.1-NR), respectively (**Figure 4**). The HR for death in LAT1-high *vs*. LAT1-low was 2.83 (95% CI: 1.40-5.74; p = 0.0025).

GSEA further elucidated the biological context of LAT1 overexpression in HNSCC (**Supplementary Material**). LAT1-high tumors exhibited significant enrichment of hypoxia-related pathways, with the HALLMARK_HYPOXIA gene set showing a strong positive enrichment score (NES = 1.88; FDR = 0.01). In parallel, the HALLMARK GLYCOLYSIS gene set was also highly enriched (NES = 1.87; FDR = 0.008), indicating an upregulation of glycolytic metabolism. Consistent findings were observed across two independent validation cohorts and were further supported by *in vitro* data, as detailed in the **Supplementary Material**.

## 4 Discussion

Our data support the role of LAT1 as a negative prognostic biomarker in HNSCC, building upon and complementing prior findings across multiple tumor types (4). In our cohort of 100 patients with locally advanced HNSCC, high LAT1 expression was associated with significantly worse overall survival, and was enriched in transcriptomic subtypes previously linked to biologically aggressive and radioresistant behavior, specifically, the mesenchymal (Cluster 2) and hypoxic (Cluster 3) subtypes. Conversely, the immune-reactive cluster (Cluster 6), known for favorable prognosis and responsiveness to immune checkpoint inhibitors (12), showed the lowest LAT1 levels.

In line with the ancillary role of CD98 (SLC3A2) in stabilizing LAT1 and enabling its transport function, we explored its expression in our cohort. CD98 and LAT1 showed a strong positive correlation (r = 0.577, p = 1.62 × 10^-10^), consistent with their known functional interdependence. Similar to LAT1, CD98 expression was highest in the hypoxic (Cluster 3) and mesenchymal (Cluster 2) subtypes, and lowest in the immune-reactive subtype (Cluster 6). This concordance not only reflects the close biological link between the two proteins, but also suggests that simultaneous assessment of LAT1 and CD98 could provide complementary insights into tumor aggressiveness. Moreover, the recent availability of cryo-EM structures of the LAT1–CD98 heterodimer offers new opportunities for rational drug design and for future studies investigating their combined role in cancer development.

Given the biological heterogeneity of HNSCC, we specifically examined LAT1 expression across both anatomical subsites and transcriptomic-defined molecular clusters to explore its distribution in clinically and biologically distinct subgroups. The co-enrichment of hypoxia and glycolysis signatures suggests that HNSCC cells undergo metabolic reprogramming to adapt to oxygen-deprived conditions, promoting tumor survival and progression. These insights highlight metabolic vulnerabilities that could be exploited for therapeutic purposes in HNSCC.

LAT1 mainly mediates the transport of essential amino acids, including leucine, isoleucine, valine, phenylalanine, tyrosine, tryptophan, histidine, and methionine. This property is central to cancer biology, since uptake of these amino acids fuels anabolic processes, activates mTORC1 signaling, and supports tumor growth under nutrient-limiting conditions. Through this metabolic rewiring, LAT1 overexpression enables tumor cells to adapt to stress and sustain proliferation, thereby linking its functional role as a nutrient gatekeeper with the aggressive phenotype observed in LAT1-high HNSCC (PMID: 25580517; PMID: 26749017; PMID: 32198649).

In the context of BNCT, LAT1’s clinical relevance becomes even more pronounced (4). LAT1 facilitates intracellular uptake of BPA, a key carrier molecule used in BNCT. Although LAT1 is not used as a clinical selection criterion for BNCT, its overexpression may indicate tumor biology that could benefit from this high-LET treatment approach. This is particularly relevant in head and neck oncology, where promising clinical results from Japan and Finland have been reported in patients with recurrent HNSCC treated with BNCT (13, 14). These results raise interest in identifying biologically enriched subgroups that may rationally be considered for BNCT in future studies, even if LAT1 alone cannot serve as a standalone selection biomarker.

An additional unique feature of LAT1 is its dual role as both a drug transporter and a therapeutic target. LAT1 can be exploited for carrier-mediated drug delivery, for instance with boronophenylalanine in BNCT or with LAT1-selective PET tracers that demonstrate high tumor uptake and favorable tumor-to-background ratios. At the same time, LAT1 itself is a druggable target, as shown by the selective inhibitor nanvuranlat (JPH203), which has entered clinical trials. This duality underscores the translational relevance of LAT1 in oncology [PMID: 26101697; PMID: 32377929; PMID: 22743251 (20)].

Preclinical and clinical data reinforce this rationale. LAT1-overexpressing models exhibit enhanced boron uptake, greater BNCT efficacy, and favorable tumor-to-background ratios using LAT1-specific PET tracers such as [^18^F]-FBPA and [^18^F]-FBY (15–18). Clinical experience in Japan (13) and Finland (14) has shown promising outcomes with BNCT in recurrent HNSCC, including high objective response (75-76%) and complete remission (50-75%) rates, which is numerically competitive if compared with response to chemotherapy or chemo-immunotherapy for the same setting [36% (19)].

Beyond BNCT, LAT1 is also being investigated as a direct therapeutic target. The LAT1 inhibitor nanvuranlat showed encouraging results in a phase 2 randomized placebo-controlled study in patients with pretreated refractory biliary tract cancer (20). The study reported a significantly improved progression-free survival (HR = 0.557) and a disease control rate of ~25% in the treatment arm. Nanvuranlat has also demonstrated anti-metastatic effects in preclinical models of melanoma (21). Additional LAT1-targeted agents, such as QBS10072S and QBS10096S, are being developed for aggressive lymphomas (22).

Finally, LAT1 expression has been linked to chemoresistance in gastric cancer (23) and cell cycle progression in pancreatic cancer through p38 MAPK activation and cyclin D1 suppression (24). Emerging evidence also suggests its role as a non-invasive biomarker via detection in exosomes (25). Further validation analyses across independent datasets and integrative functional investigations are presented in the **Supplementary Material**, confirming the reproducibility and biological relevance of our findings.

A limitation of this study is its retrospective nature and the single-institution design, which may limit the generalizability of our findings. In addition, LAT1 expression was assessed at the transcriptomic level without corresponding protein validation. Acknowledging these limitations, our data indicate that high LAT1 expression in HNSCC is a negative prognostic factor, and LAT1 is enriched in radioresistant transcriptional subtypes (Cl2 Mesenchymal, Cl3 Hypoxia). These findings are consistent with previously published evidence (26, 27).

While LAT1 is not currently used for clinical selection of BNCT candidates, its biological profile overlaps with tumor characteristics that may benefit from this therapeutic strategy. Further prospective studies will be needed to clarify the role of LAT1 in guiding BNCT or other therapeutic approaches in HNSCC, particularly in light of the favorable outcomes reported in early-phase BNCT studies.

## Data Availability

The original contributions presented in the study are publicly available. This data can be found on the Gene Expression Omnibus repository (GSE313289).
