# Supplementary material for: LAT1 expression in head and neck cancer: a prognostic biomarker with potential relevance for BNCT
Source: Front Oncol. 2025 Dec 19;15:1678011. doi: 10.3389/fonc.2025.1678011 (PMC12757229; doi:10.3389/fonc.2025.1678011)
Supplement: Supplementary file 1 [file DataSheet1.docx]

Supplementary Material

# Supplementary Data

## External validation

To validate LAT1 expression and its prognostic significance in an independent cohort of HNSCC patients, we performed external validation analyses using two additional, independent datasets: a second BD2Decide cohort (BD2 cohort2) and the TCGA-HNSCC dataset. The initial BD2Decide cohort (here referred to as BD2 cohort1) served as the primary testing set, designed to include balanced representation across the major head and neck anatomical subsites—oral cavity, oropharynx (both HPV-positive and HPV-negative), hypopharynx, and larynx. This cohort thus reflects the biological and clinical heterogeneity of HNSCC. LAT1 expression was measured from transcriptomic data and stratified into LAT1-high and LAT1-low groups.

In BD2 cohort1, high LAT1 expression was significantly associated with inferior overall survival. As shown in the Kaplan–Meier plot, LAT1-high patients displayed a marked decrease in survival probability compared with LAT1-low patients (p = 0.0024). The estimated hazard ratio (HR) for death was 2.83 (95% CI 1.40–5.74; p = 0.0025), confirming LAT1 as a strong and independent negative prognostic marker in this real-world, radiotherapy-treated population.

To assess reproducibility, we validated these findings in an independent Italian cohort (BD2 cohort2) composed of patients treated at different institutions but with comparable clinical and molecular annotation. Consistent with the discovery data, LAT1-high tumors showed significantly worse overall survival, as illustrated in the corresponding Kaplan–Meier plot (Supplementary Figure 2), with an HR = 2.84 (95% CI 1.14–7.04; p = 0.002). The parallel magnitude and direction of effect between cohort1 and cohort2 confirm the robustness and reproducibility of LAT1’s prognostic role across independent datasets. Importantly, both analyses were adjusted for potential confounding factors including age, performance status, primary site, and HPV status, confirming that the association between LAT1 expression and survival was independent of standard clinical parameters.

To further strengthen this observation, LAT1 expression was evaluated in the TCGA-HNSCC dataset, an external, publicly available cohort with RNA-sequencing data and comprehensive clinical annotation. Kaplan–Meier analysis (Supplementary Figure 3) again demonstrated significantly reduced overall survival in LAT1-high patients (p = 0.0037), with an HR = 1.49 (95% CI 1.14–1.94). This effect size, though slightly attenuated compared to the BD2 cohorts, remains statistically significant and directionally consistent, validating LAT1’s adverse prognostic impact in an entirely independent patient population analyzed through a different transcriptomic platform.

The consistency of these findings across three independent cohorts—two institutional microarray datasets and one TCGA RNA-seq dataset—strongly supports the reproducibility of LAT1 as a negative prognostic biomarker in head and neck squamous cell carcinoma. Furthermore, the overlap between the BD2 and TCGA datasets in terms of transcriptomic signatures reinforces the biological coherence of LAT1-associated pathways. LAT1-high tumors in all cohorts were enriched in hypoxia and glycolysis gene sets, consistent with metabolic reprogramming and radioresistance, whereas LAT1-low tumors exhibited immune-related gene-expression features and better prognosis.

In summary, the prognostic significance of LAT1 expression has been validated across three independent HNSCC cohorts, encompassing diverse anatomical subsites, treatment modalities, and transcriptomic platforms. The hazard ratios remained consistently above 1.4, and all p-values were statistically significant (BD2 cohort1: HR = 2.83, p = 0.0025; BD2 cohort2: HR = 2.84, p = 0.002; TCGA: HR = 1.49, p = 0.0037). These results confirm that LAT1 overexpression reproducibly identifies a biologically aggressive subgroup of HNSCC with unfavorable prognosis. Collectively, this multi-cohort validation fulfills the reviewer’s request by demonstrating both technical reproducibility and biological consistency of LAT1 as a prognostic biomarker in head and neck cancer.

## Functional in vitro studies

SLC7A5, also known as LAT1, was discovered as a transporter in activated lymphocytes in 1992 and was later identified as a heterodimeric complex in humans in 1998. Its history is linked to understanding how cells take in essential amino acids, its role in conditions like cancer, and its significance in development and other physiological processes. Considering its biological role, functional in vitro experiments targeting LAT1 in HNSCC have been explored in many studies suggesting that SLC7A5-mediated leucine influx sustains mTORC1 signaling, metabolic fitness, and stress tolerance, thereby enhancing clonogenic survival after irradiation and promoting invasion. Direct LAT1 knockdown/overexpression data in HNSCC cell lines are reported and convergent evidence supports that LAT1 activity is clinically actionable and measurable via the LAT1-selective PET tracer 18F‑FAMT, whose uptake correlates with LAT1 expression and prognosis, underscoring the transporter’s tumor-biologic relevance and translational tractability [1]. At the pathway level, YAP/TAZ drive SLC7A5 transcription, increasing amino‑acid uptake and activating mTORC1; pharmacologic or genetic disruption of this axis suppresses proliferation, establishing a causal link between LAT1 and growth signaling that is likely conserved across epithelial cancers [2]. Canonical transport biology further validates LAT1’s role in essential amino‑acid transport critical for biosynthesis and signaling, providing assayable endpoints (leucine uptake, p70S6K/4E‑BP1 phosphorylation) for functional studies in HNSCC lines [3][4]. Radiobiology models in HNSCC demonstrate that intrinsic radioresponse is shaped by tumor-intrinsic programs and microenvironmental context: FaDu exhibits dose‑dependent survival loss with pathway modulation after X‑rays, providing a suitable platform for SLC7A5 silencing to test radiosensitization via clonogenic assays, γH2AX foci, and apoptosis readouts [5]; 3D spheroids from HPV‑negative CAL27 and HPV‑positive comparators retain in vivo–like radiosensitivity gradients, enabling evaluation of LAT1 manipulation under metabolically stressed, hypoxic architectures where amino‑acid competition is most pronounced [6]. More broadly, metabolic reprogramming and nutrient competition in the tumor–immune ecosystem link amino‑acid transport to acidosis, immune dysfunction, and therapeutic resistance, offering additional phenotypes (lactate, pH, cytokines) to quantify LAT1‑dependent aggressiveness and RT tolerance in vitro [7]. Collectively, these data justify HNSCC cell line experiments using siRNA/CRISPR SLC7A5 knockdown or cDNA overexpression, plus LAT1 blockade, to test effects on mTORC1 signaling, migration/invasion, and radiation response, anchored to LAT1’s validated imaging surrogate and conserved upstream regulators.

[1] Oriuchi, N., et al. Clinical significance of 18F-alpha-methyl tyrosine PET as a potential surrogate marker of LAT1 expression for predicting prognosis of non-small cell lung cancer. Journal of Clinical Oncology, 2010, 28: 10559-10559. https://doi.org/10.1200/JCO.2010.28.15_SUPPL.10559

[2] Park, Yun‐Yong, et al. Yes‐associated protein 1 and transcriptional coactivator with PDZ‐binding motif activate the mammalian target of rapamycin complex 1 pathway by regulating amino acid transporters in hepatocellular carcinoma. Hepatology, 2016, 63. https://doi.org/10.1002/hep.28223

[3] Palacin, M., et al. Molecular biology of mammalian plasma membrane amino acid transporters. Physiological Reviews, 1998, 78 4: 969-1054. https://doi.org/10.1152/PHYSREV.1998.78.4.969

[4] Malandro, M. Molecular biology of mammalian amino acid transporters. Annual Review of Biochemistry, 1996, 65: 305-36. https://doi.org/10.1146/ANNUREV.BI.65.070196.001513

[5] Duarte, A., et al. PO-275 Effect of ionising radiation in FaDu cell line- preliminary results. Esmo Open, 2018, 3: 128-128. https://doi.org/10.1136/esmoopen-2018-EACR25.306

[6] Wegge, M., et al. Use of 3D Spheroid Models for the Assessment of RT Response in Head and Neck Cancer. International Journal of Molecular Sciences, 2023, 24. https://doi.org/10.3390/ijms24043763

[7] Xia, Longzheng, et al. The cancer metabolic reprogramming and immune response. Molecular Cancer, 2021, 20. https://doi.org/10.1186/s12943-021-01316-8

## Integration with additional molecular and clinical biomarkers

The indication for biomarker integration can differ substantially depending on whether the focus is on locally advanced, radio(chemo)therapy-treated disease, or on recurrent/metastatic (R/M) HNSCC managed with systemic agents such as immunotherapy.

LAT1 (L-type amino acid transporter 1) has been implicated in tumor metabolism, proliferation, and therapy resistance, and its role may vary significantly across clinical settings. In the context of definitive radiotherapy or concurrent chemoradiation, LAT1 overexpression could indeed be associated with hypoxia-driven metabolic reprogramming and radioresistance. In the present manuscript, LAT1 expression was already investigated in patients with locally advanced HNSCC treated with multimodal therapy including radiotherapy, as part of the BD2Decide project. In that study, we applied the six-cluster molecular classification described by De Cecco et al. (Oncotarget 2015;6:9627–42) and demonstrated that LAT1 expression significantly varied across these transcriptomic subtypes. LAT1 was highest in the mesenchymal (Cl2) and hypoxic (Cl3) clusters, both associated with poor prognosis and radioresistance, while it was lowest in the immune-reactive (Cl6) subtype, which is linked to better outcomes and higher radiosensitivity. These findings, in line with GSEA results showing enrichment of hypoxia and glycolysis pathways in LAT1-high tumors, already addressed the association of LAT1 with molecular features relevant to radiotherapy resistance. Therefore, the integration of LAT1 with hypoxia signatures and molecular subtypes has already been accomplished in the context of locally advanced, curatively treated HNSCC.

In contrast, for patients with recurrent or metastatic disease, LAT1 expression may instead reflect immune-metabolic features and aggressiveness of tumors that have already escaped prior local therapy. Therefore, the proposed integration of LAT1 with p53 or hypoxia markers would be most relevant for the radiotherapy setting, whereas PD-L1 expression and immune-related signatures are instead meaningful for the immunotherapy-treated population. Given that PD-L1 is currently the only clinically approved biomarker for treatment selection in R/M HNSCC receiving immune checkpoint inhibitors (ICIs) such as nivolumab or pembrolizumab, we decided to specifically explore LAT1 expression in the NIVACTOR study.

The NIVACTOR study (“A Single-Arm, Open-Label, Multicenter, Phase IIIb Clinical Trial with Nivolumab in Subjects with Recurrent or Metastatic Platinum-Refractory Squamous Cell Carcinoma of the Head and Neck”) was a pivotal real-world investigation designed to assess the clinical efficacy, safety, and molecular predictors of response to the anti–PD-1 antibody nivolumab in patients with recurrent or metastatic head and neck squamous cell carcinoma (HNSCC) who had relapsed or progressed following platinum-based therapy. The trial was approved by the Italian Drug Agency (AIFA) in 2017 under EudraCT number 2017-000562-30 and conducted across twenty-one oncology centers in Italy. Between November 2017 and July 2018, 127 patients were enrolled, and 124 received treatment, representing a broad and clinically heterogeneous cohort reflecting the real-world landscape of this challenging disease setting.

Nivolumab was administered at a fixed dose of 240 mg intravenously every two weeks until disease progression, unacceptable toxicity, death, or withdrawal of consent. Eligible patients had recurrent or metastatic disease that was refractory to platinum therapy, defined as progression within six months after completion of chemoradiation or failure of first-line systemic therapy. Prior radiation or cetuximab-based treatment was permitted. Tumor response and progression were assessed according to RECIST version 1.1 criteria by local investigators. The primary endpoint of the study was the incidence of high-grade (grade 3 or higher) treatment-related adverse events, assessed according to the CTCAE version 4.03. Secondary endpoints included overall survival (OS), progression-free survival (PFS), objective response rate (ORR), and disease control rate (DCR). OS was defined as the time from the first nivolumab dose to death from any cause, and PFS as the time from treatment initiation to radiographic or clinical progression or death. The ORR was calculated as the proportion of patients who achieved a complete or partial response, while the DCR included patients who obtained complete response, partial response, or stable disease. Exploratory endpoints focused on the identification of biomarkers associated with response and survival, particularly the evaluation of PD-L1 expression, tumor mutational burden (TMB), and immune-related gene-expression signatures.

The NIVACTOR trial confirmed nivolumab’s safety and efficacy in a population of patients representative of everyday clinical practice. Most participants were male (81%) with a median age of 64 years, and the primary tumor sites were distributed across the oral cavity (33%), oropharynx (23%), and larynx (25%). The majority of patients (86%) had received prior radiotherapy. Nivolumab demonstrated a favorable safety profile consistent with earlier clinical trials, with manageable immune-related adverse events and no unexpected toxicities. The incidence of grade ≥3 treatment-related events was in line with the established tolerability of PD-1 inhibitors.

Clinically, nivolumab provided meaningful benefit in a subset of patients. Responders, those achieving complete or partial response, showed a striking improvement in overall survival compared to non-responders. Median OS was not reached in responders, whereas it was 4.6 months in non-responders, yielding a hazard ratio of 9.98 (p<0.00001). Patients who achieved disease control (complete response, partial response, or stable disease) also experienced significantly better outcomes, with a median OS of 17.8 months compared with 3.8 months for patients with progressive disease. Similarly, progression-free survival was markedly improved among disease-controlled patients, with a median PFS of 9.3 months versus 2.0 months in those with progression. These data highlighted the durable benefit achievable with nivolumab in a subset of HNSCC patients, reinforcing its role as a standard therapy in the post-platinum setting.

To explore biological determinants of response, the study included an extensive translational research program. Formalin-fixed paraffin-embedded (FFPE) tumor samples were analyzed for PD-L1 expression, tumor mutational burden, and transcriptomic signatures. PD-L1 was assessed using the 22C3 pharmDx assay, with both tumor proportion score (TPS) and combined positive score (CPS) evaluated. Among patients with available tissue, those with a CPS ≥1 demonstrated significantly longer overall survival compared with PD-L1–negative cases, with a median OS of 7.4 months versus 4.8 months (HR=1.75; p=0.04). However, progression-free survival differences did not reach significance. High TPS (≥50%) was rare, found in only six patients, but tended to associate with longer OS (16.4 versus 6.8 months), suggesting that higher PD-L1 expression may enrich for benefit though not serving as an exclusive predictor. Overall, PD-L1 showed a modest predictive role, echoing the limited sensitivity and specificity observed in other studies of HNSCC immunotherapy.

Tumor mutational burden (that includes and expand the basic p53 status) was determined using the Illumina TruSight Oncology 500 panel. Samples with at least 10 mutations per megabase were categorized as TMB-high. Among 63 evaluable patients, only seven fell into this category. The analysis revealed no significant association between TMB status and either clinical response or survival. The median OS was 11.0 months for high-TMB patients compared to 7.4 months for low-TMB, and hazard ratios were not statistically significant for either OS (HR=0.7) or PFS (HR=0.82). This lack of correlation indicates that, in head and neck cancer, mutational burden alone does not effectively capture the immunogenic potential of tumors or predict benefit from PD-1 blockade. These findings are consistent with other recent evidence suggesting that the immune microenvironment, rather than genomic mutation rate, is the dominant determinant of response to immune checkpoint inhibitors in HNSCC.

In the NIVACTOR cohort of recurrent/metastatic HNSCC treated with nivolumab, LAT1 expression emerged as a potential biomarker of outcome. The Kaplan–Meier survival analysis (Supplementary Figure 4) demonstrates that patients with high LAT1 expression experienced significantly shorter overall survival compared with those with low LAT1 levels (p = 0.025). The survival curves diverge early during treatment, indicating that LAT1-high tumors may possess intrinsic resistance mechanisms that limit the long-term benefit of PD-1 blockade. This finding aligns with LAT1’s biological role as a metabolic transporter linked to tumor proliferation, hypoxia adaptation, and immune evasion.

The corresponding clinical and molecular characteristics table confirms that LAT1-high and LAT1-low groups were balanced across key demographic and biological variables. No significant differences were observed in sex (p = 0.99), smoking status (p = 0.99), or ECOG performance status (p = 0.801), excluding clinical confounding as the cause of survival differences. Importantly, LAT1 expression was not correlated with PD-L1 (CPS ≥1, p = 0.243) or tumor mutational burden (TMB >10, p = 0.166), suggesting that LAT1 identifies a biologically distinct subset independent of established immunotherapy biomarkers.

Overall, these results indicate that LAT1 overexpression marks a metabolically aggressive, immune-resistant phenotype in R/M HNSCC, potentially associated with reduced nivolumab efficacy. This supports further investigation of LAT1 as an immunometabolic biomarker and as a possible therapeutic target to overcome resistance to PD-1 inhibition.

A major strength of the NIVACTOR study was the comprehensive gene-expression profiling performed on a subset of 80 patients. RNA extracted from FFPE material was hybridized on Clariom D microarrays, interrogating over 540,000 transcripts. The analysis included thirteen predefined immune-related gene-expression signatures previously associated with response or prognosis in immunotherapy-treated cancers. Among these, the IFNγ-expanded signature, first reported by Ayers et al. in 2017, emerged as a key biomarker of sensitivity to nivolumab. This signature comprises 18 genes linked to interferon-γ signaling and cytotoxic immune activation, including IDO1, CXCL9, CXCL10, STAT1, HLA-DRA, CD3E, GZMB, and LAG3. In NIVACTOR, patients with high IFNγ-expanded scores demonstrated improved overall and progression-free survival, reflecting a transcriptionally “inflamed” tumor phenotype characterized by T-cell infiltration, antigen presentation, and interferon-driven immune activation. These results reinforce the concept that tumors with pre-existing immune engagement are more likely to respond to PD-1 blockade.

In contrast, the Chr9 loss signature, derived from genomic analyses of chromosome 9p deletions in HNSCC, was associated with poor outcomes. This signature encompasses genes involved in immune regulation and cell cycle control, including JAK2, CDKN2A, IFNE, and IL33. NIVACTOR demonstrated that patients with high Chr9-loss scores had significantly worse survival: median OS was 3.3 months for high-score tumors versus 8.7 months for low-score tumors (HR=3.5, p=0.0003). Similarly, progression-free survival was shorter (2.0 versus 2.2 months, HR=2.4, p=0.012). These findings suggest that loss of chromosomal integrity in this region disrupts interferon signaling and antigen presentation pathways, leading to immune escape and resistance to nivolumab.

Interferon‑γ (IFN‑γ) orchestrates antitumor immunity in HNSCC by enhancing antigen presentation and inducing PD‑L1, thereby shaping responsiveness to PD‑1/PD‑L1 blockade; these effects are observed both in tumor tissue and peripherally when IFN‑γ is administered with nivolumab. However, PD‑L1 heterogeneity and the imperfect predictive value of PD‑L1 alone highlight IFN‑γ paradox: the same pathway that drives immune recognition can also result in adaptive resistance via PD‑L1 upregulation and an inflamed gene‑expression program (T‑cell‑inflamed GEP) that only partially overlaps with genomic predictors such as TMB. Tumor‑intrinsic barriers modulate IFN‑γ efficacy: hypoxia curtails T‑cell function and associates with inferior anti‑PD‑1 outcome in HNSCC, suggesting that IFN‑γ signaling operates within, and can be blunted by metabolic and stromal constraints. Smoking further suppresses IFN response programs and cytotoxic infiltration, potentially diminishing IFN‑γ driven tumor control. Metabolically, IFN‑γ activity unfolds in a nutrient‑limited ecosystem marked by lactate, acidosis, and amino‑acid competition; these features favor tumor survival pathways converging on PI3K–AKT–mTOR and can impose functional attrition on effector lymphocytes. In this context, LAT1 (SLC7A5) dependent leucine influx and mTORC1 activation plausibly sustain tumor growth under IFN‑γ mediated stress and reinforce immune evasion, aligning with evidence that dampening upstream inputs to mTOR (for example, via HER3 blockade) reconditions the microenvironment and augments PD‑1 therapy. Clinically, these dynamics help explain why PD‑1/PD‑L1 inhibitors benefit a subset, often those with concurrent inflamed transcriptional states, while others exhibit early progression or hyperprogression and only later respond to chemotherapy. Therapeutically, integrating IFN‑γ‑axis biomarkers (PD‑L1, T‑cell‑inflamed), metabolic context (hypoxia), and mTOR‑linked signaling may better stratify patients and nominate combinations that pair immunotherapy with metabolic or upstream RTK interventions to counteract IFN‑γ conditioned resistance. In the NIVACTOR study, tumors with high LAT1 expression show significantly higher Chr9 loss signature scores compared with LAT1-low tumors (p = 0.00118). This indicates that LAT1 overexpression is associated with transcriptional features linked to chromosome 9p deletion, a genomic alteration involving genes such as JAK2, CDKN2A, and IFNE, which are related to immune escape and therapy resistance. The correlation supports the hypothesis that LAT1-high tumors exhibit a genetically unstable, radio- and immune-resistant phenotype.

LAT1-high tumors also display significantly higher IFNγ-expanded signature scores (p = 0.0333). The IFNγ-expanded signature reflects activation of interferon-γ signaling and the presence of a T-cell–inflamed tumor microenvironment. This indicates that LAT1-high tumors, despite their aggressive biology, retain a transcriptionally active immune contexture, potentially characterized by immune stimulation alongside immune exhaustion. These findings suggest a complex interplay between metabolic activation and immune signaling, where LAT1 overexpression may coexist with an inflammatory yet dysfunctional immune phenotype.

Overall, the NIVACTOR study provides compelling evidence that clinical benefit from nivolumab in platinum-refractory head and neck cancer is driven more by the immune transcriptional landscape than by PD-L1 or TMB alone. While PD-L1 retains partial prognostic value and TMB remains uninformative, interferon-related gene signatures such as IFNγ-expanded serve as robust indicators of microenvironment context, whereas genomic alterations such as Chr9 loss define a subgroup with immune-cold, therapy-resistant biology. The integration of these findings highlights the complexity of immunotherapy response in HNSCC and underscores the need for multi-dimensional biomarkers that capture both immune activation and genomic context. NIVACTOR thus stands as one of the most comprehensive real-world analyses linking immunotherapy outcomes with detailed molecular correlates, offering important insights for patient stratification and the future personalization of PD-1 blockade in head and neck cancer.

# Supplementary Figures and Tables

## Supplementary Figures


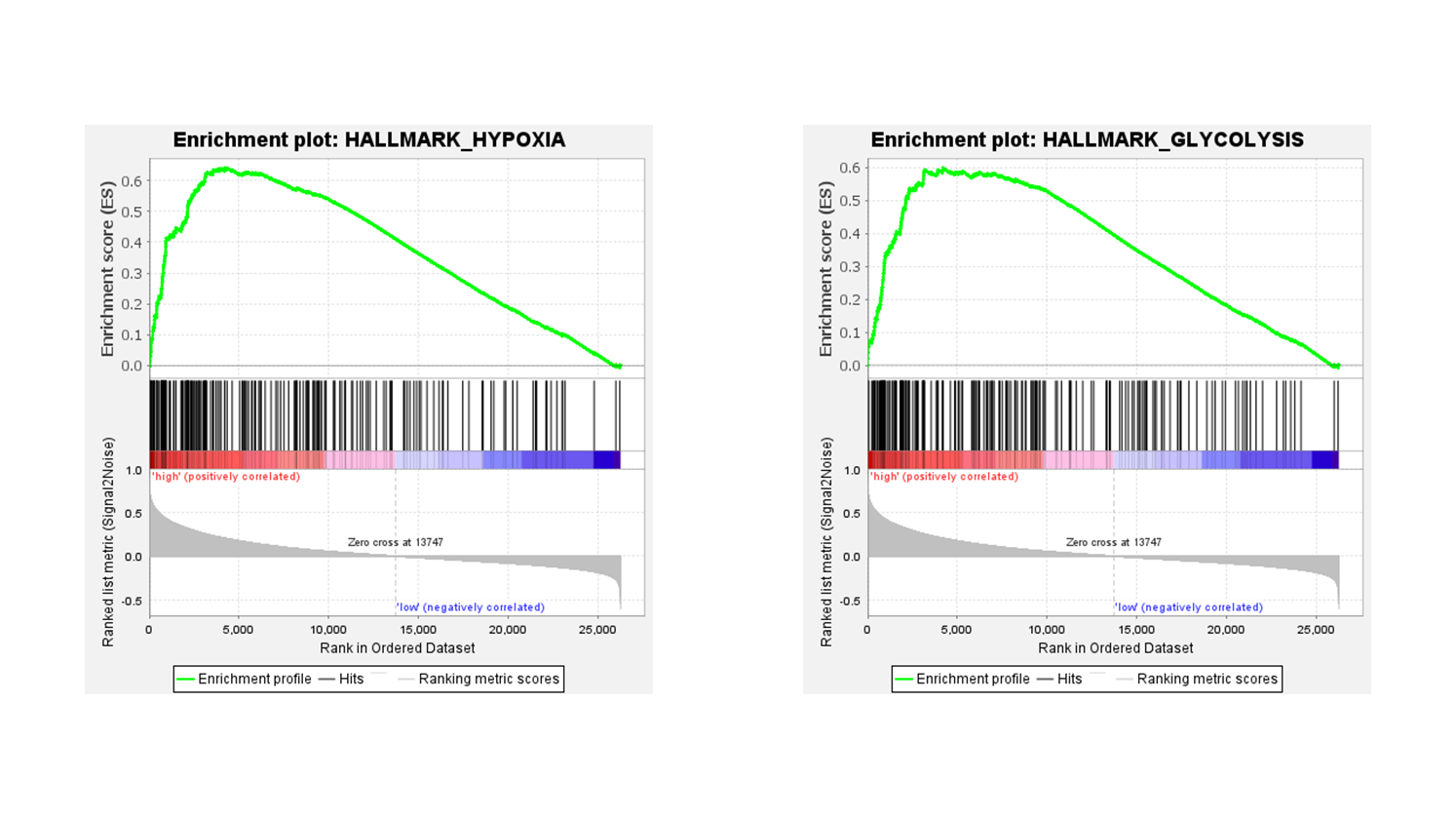


**Supplementary Figure 1.** Hypoxia and glycolysis pathways assessed through Gene Set Enrichment Analysis (GSEA) in LAT1-high vs. low patients

**Supplementary Figure 2.** LAT1 expression in the BD2 cohort 2 dataset

**Supplementary Figure 3.** LAT1 expression in the TCGA-HNSCC dataset

**Supplementary Figure 4.** LAT1 expression in the Nivactor dataset
